# Desulfination by 2′-hydroxybiphenyl-2-sulfinate desulfinase proceeds *via* electrophilic aromatic substitution by the cysteine-27 proton†
†Electronic supplementary information (ESI) available: Molecular dynamics simulations method, additional data, equations, and Cartesian coordinates of active site cluster models. See DOI: 10.1039/c7sc00496f


**DOI:** 10.1039/c7sc00496f

**Published:** 2017-05-17

**Authors:** Inacrist Geronimo, Shawn R. Nigam, Christina M. Payne

**Affiliations:** a Department of Chemical and Materials Engineering , University of Kentucky , Lexington , Kentucky 40506-0046 , USA . Email: christy.payne@uky.edu

## Abstract

Biodesulfurization is an attractive option for enzymatically removing sulfur from the recalcitrant thiophenic derivatives that comprise the majority of organosulfur compounds remaining in hydrotreated petroleum products. Desulfurization in the bacteria *Rhodococcus erythropolis* follows a four-step pathway culminating in C–S bond cleavage in the 2′-hydroxybiphenyl-2-sulfinate (HBPS) intermediate to yield 2-hydroxybiphenyl and bisulfite. The reaction, catalyzed by 2′-hydroxybiphenyl-2-sulfinate desulfinase (DszB), is the rate-limiting step and also the least understood, as experimental evidence points to a mechanism unlike that of other desulfinases. On the basis of structural and biochemical evidence, two possible mechanisms have been proposed: nucleophilic addition and electrophilic aromatic substitution. Density functional theory calculations showed that electrophilic substitution by a proton is the lower energy pathway and is consistent with previous kinetic and site-directed mutagenesis studies. C27 transfers its proton to HBPS, leading directly to the release of SO_2_ without the formation of a carbocation intermediate. The H60–S25 dyad stabilizes the transition state by withdrawing the developing negative charge on cysteine. Establishing the desulfination mechanism and specific role of active site residues, accomplished in this study, is essential to protein engineering efforts to increase DszB catalytic activity, which is currently too low for industrial-scale application.

## Abstract 2

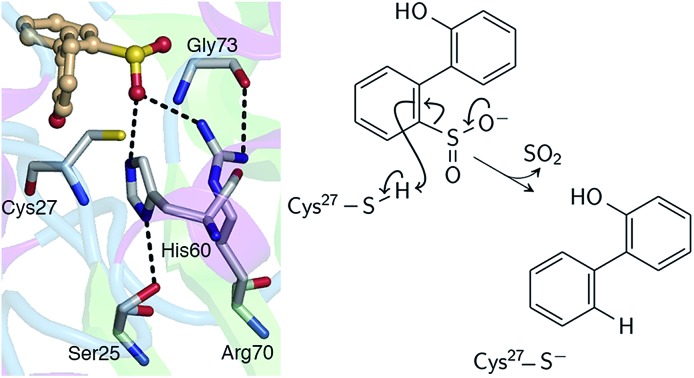
Density functional theory shows that the rate-limiting desulfination step in biodesulfurization involves concerted electrophilic substitution with the Cys-27 proton.

## Introduction

Declining reserves of low-impurity crude oil and increasingly strict environmental regulations on transportation fuel sulfur content has stimulated biochemical and genetic research on biodesulfurization in recent years.^1^–^3^ This process is an attractive complementary method to conventional hydrodesulfurization for upgrading heavy, sulfur-rich crude oil because it degrades dibenzothiophene (DBT) and its derivatives, selectively removes sulfur without diminishing the calorific value of the product, and can be conducted safely under ambient conditions.^3^,^4^ Biodesulfurization of DBT by *Rhodococcus erythropolis* is accomplished in a four-step pathway by four different enzymes. Sulfur is liberated from 2′-hydroxybiphenyl-2-sulfinate (HBPS) in the final step, where it is detected as HSO_3_^–^ (Scheme S1†).^4^–^6^ This desulfination step, catalyzed by 2′-hydroxybiphenyl-2-sulfinic acid desulfinase (DszB), has the lowest reaction rate^4^ and is, therefore, a major bottleneck in the biodesulfurization process.^7^

Structural and biochemical studies on DszB have been unable to resolve its reaction mechanism, hindering protein engineering efforts to enhance enzyme activity. DszB is also interesting from a purely biochemical perspective because experimental studies suggest that it has evolved to employ a unique desulfurization mechanism. Unlike the other enzymes that catalyze desulfination, cysteine sulfinate desulfinase (CSD)^8^ and l-aspartate β-decarboxylase,^9^ DszB is not assisted by pyridoxal 5′-phosphate or any other cofactor.^4^,^10^

**Fig. 1:**
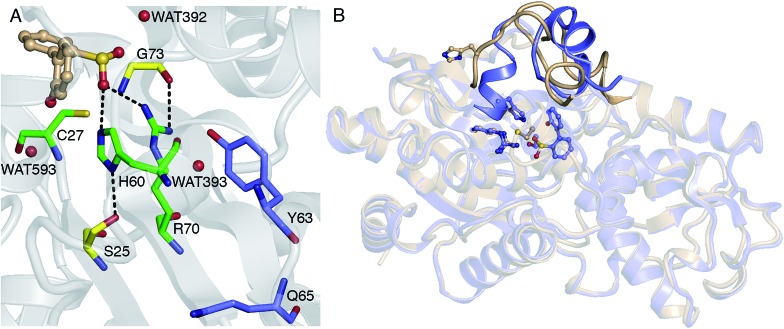
(A) Active site model based on the crystal structure of the C27S DszB mutant in complex with 2′-hydroxybiphenyl-2-sulfinate (HBPS, tan) (PDB entry 2DE3 (ref. 12)). C27, H60, and R70 (green) have been implicated in desulfination. S25 and G73 (yellow), and crystallographic water molecules (red spheres) hydrogen bonded to HBPS and R70 were also included in the computational model. Y63 and Q65 (violet) are not involved in the reaction, but their mutation was observed to affect activity.^13^ (B) Superimposed structures of substrate-free wild-type DszB (PDB entry ; 2DE2,^12^ tan) and C27S mutant (violet). In the latter, residues 55–62 and 187–204 (shown with less transparency) form α-helices in the presence of HBPS, causing entry of H60 (in ball-and-stick representation) into the active site.

Thus far, it has been established that C27 is critical to activity based on inhibition by Cu^2+^, Zn^2+^, and cysteine-modifying reagents,^10^,^11^ and inactivation upon mutation to serine.^10^,^12^ H60 and R70 are also thought to be involved in the reaction, as these residues appear to form hydrogen bonds with HBPS in the C27S mutant crystal structure (Protein Data Bank entry ; 2DE3 (ref. 12)) (Fig. 1A). H60 is introduced into the active site upon substrate binding, when the loops composed of residues 55–62 and 187–204 in the native enzyme (PDB entry ; 2DE2 (ref. 12)) form α-helices (Fig. 1B). However, mutation of H60 to glutamine only reduced activity by ∼17 fold.^12^ R70I and R70K mutants produced in the same study were present in the insoluble fraction of cell extracts, which did not exhibit detectable desulfination activity. R70 is part of a highly conserved RXGG motif found in homologs of DszB and was, thus, assumed to play a structural role.^12^

**Scheme 1:**
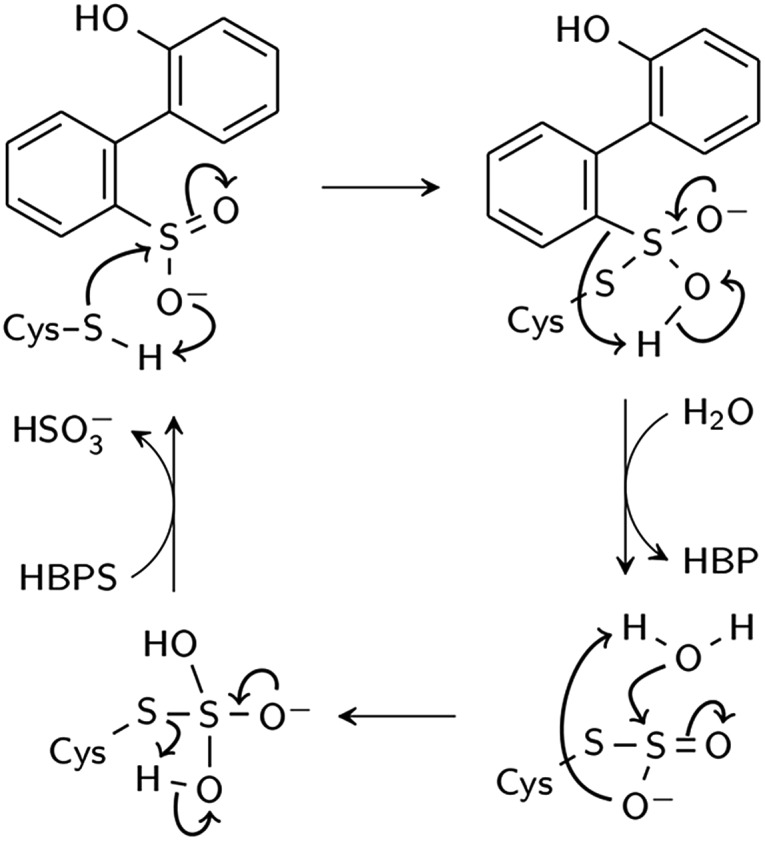
Proposed nucleophilic addition mechanism (Lee *et al.*^12^).

**Scheme 2:**
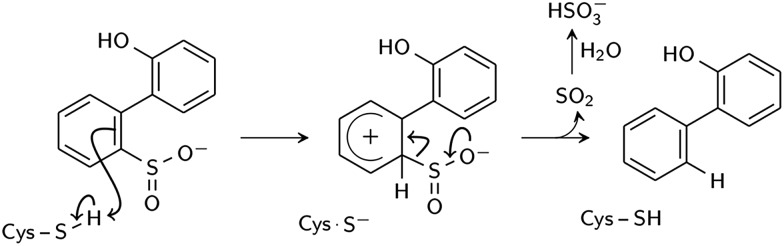
Proposed electrophilic aromatic substitution mechanism (Gray *et al.*^16^).

Two reaction mechanisms have been proposed in the literature. Lee, *et al.*, working from their ; 2DE3 crystal structure, proposed a mechanism involving nucleophilic attack on the sulfinate sulfur by C27 to break the C–S bond and form a thiosulfonate-like intermediate (Scheme 1).^12^ The sulfinate group itself would serve as the general base that activates cysteine, similar to the role of histidine in the Cys–His ion pair of cysteine proteases,^14^ since H60 is not close enough to C27. Bisulfite is subsequently released by hydrolysis. Prior to structural resolution of DszB, Gray, *et al.* also proposed a mechanism modeled after that of tyrosine phenol-lyase,^15^ involving electrophilic substitution of the sulfinate group by the C27 proton.^16^ The released SO_2_ then reacts with H_2_O to form HSO_3_^–^ and H^+^ (Scheme 2). The higher reactivity of alkyl-substituted HBPS supports this mechanism; an alkyl group at the *ortho* or *para* position would stabilize the putative carbocation intermediate.^16^

In this study, we determined the most feasible reaction pathway through molecular dynamics (MD) simulations and density functional theory (DFT) calculations. The catalytic role of residues and water molecules in the active site was investigated, as well as the effects of residue protonation state, C27S mutation, and hydroxyl substituent on HBPS on the reaction barrier.

## Results and discussion

Hypothesized desulfination mechanisms (Schemes 1 and 2) were investigated using the cluster model approach, wherein active site residues likely to participate in catalysis are chosen and treated quantum mechanically to determine enzymatic reaction properties.^17^ Cluster modeling is a computationally efficient means to test different mechanisms and rule out those that are energetically unfeasible; this approach also serves as an important first step toward hybrid quantum mechanics/molecular mechanics (QM/MM) determinations of energetic reaction barriers, which improve upon accuracy through the representation of protein dynamics that may contribute to the catalytic event. The cluster model of DszB consists of HBPS and the side chains of C27, S25, H60, R70, and G73, hereafter referred to as Model 0. The residues were selected on the basis of the X-ray crystallography and site-directed mutagenesis studies discussed in the Introduction. Optimum substrate and residue orientations for each modeled pathway were obtained by running short MD simulations (Fig. S1A†). Gas-phase DFT calculations were then performed using the B3LYP functional, which generally provides good results for diverse types of enzymatic reactions.^18^ After determining the most likely mechanism from the calculated energetic barriers, the roles of individual active site residues and water molecules were examined. For comparison, the reaction was modeled using additional functionals that have been used for a similar mechanism.^19^,^20^ Finally, limitations of the cluster model in reproducing experimental kinetic parameters are discussed.

### Nucleophilic addition mechanism

**Fig. 2:**
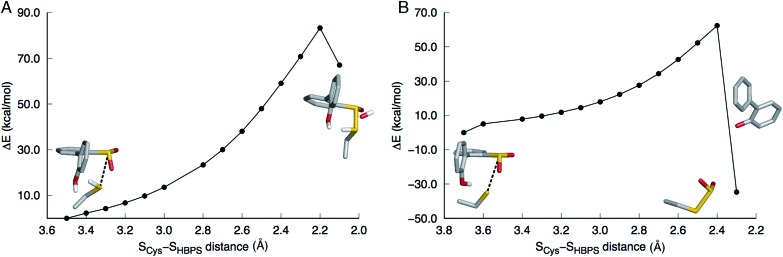
Potential energy surface scans along the S_Cys_–S_HBPS_ reaction coordinate. The model includes 2′-hydroxybiphenyl-2-sulfinate, C27, H60, R70, G73, and S25 but only the substrate and C27 are shown in stick here. (A) When C27 is modeled as protonated, the transition state is at 2.2 Å. One of the sulfinate oxygen atoms abstracts a proton from R70 and dissociates as the S–S bond is formed. (B) When C27 is modeled as deprotonated, the transition state is at 2.4 Å. The hydroxyl proton is transferred to the aromatic carbon upon C–SO_2_ bond cleavage.

In the first step of this mechanism, the protonated cysteine sulfur may either attack the sulfinate group of HBPS or initially transfer its proton to another group. An MD simulation of the enzyme with neutral C27 and negatively charged HBPS was performed to model concerted nucleophilic attack and proton transfer. Fig. S2A† shows the distribution of S–S distance with the highest frequency at 4.1 Å. The structure with the shortest S–S distance (3.5 Å) was selected for the potential energy surface (PES) scan. The transition state is located at *d*_S–S_ = 2.2 Å with an energy of 83 kcal mol^–1^. The proposed thiosulfonate-like intermediate (Scheme 1) was not formed. Instead, one of the sulfinate oxygen atoms abstracts a proton from arginine and dissociates as the S–S bond is formed (*d*_S–S_ = 2.1 Å). Cysteine also remains protonated in the resulting intermediate (Fig. 2A).

Regarding the possibility that C27 transfers its proton prior to nucleophilic attack, Lee, *et al.* noted that H60 could not be the acceptor because the S(O)-γ atom of C(S)27 and the N-ε atom of H60 are 17 and 4 Å apart in the substrate-free (; 2DE2) and substrate-bound (; 2DE3) forms, respectively.^12^ Moreover, in the substrate-bound enzyme, N-ε is more likely the protonated nitrogen since N-δ is hydrogen bonded to the S25 hydroxyl group. The retention of desulfination activity by the H60Q mutant was considered as further evidence that H60 does not act as a general base.^12^ The possible proton acceptors are, therefore, a water molecule or, as suggested by Lee, *et al.*,^12^ the sulfinate group of the substrate itself. GTPases were cited as a precedent for such substrate-assisted mechanism.^21^ An enzyme model with deprotonated C27 and negatively charged HBPS was built to represent the case where C27 has transferred its proton to the solvent. Most S–S distances fall around 4.5 Å during the MD simulation (Fig. S2B†). The PES scan along the S_Cys_–S_HBPS_ reaction coordinate was performed starting with the structure having a bond distance of 3.7 Å. The transition state is located at *d*_S–S_ = 2.4 Å with an energy of 62 kcal mol^–1^. Optimization at the next point, *d*_S–S_ = 2.3 Å, led to cleavage of the C–SO_2_ bond in HBPS and transfer of the hydroxyl hydrogen to this carbon (Fig. 2B).

The low p*K*_a_ of benzenesulfinic acid (reported experimental value varies between 1.2 and 2.8 (ref. 22–25)) suggests that HBPS is a very weak base. Nevertheless, the case wherein the sulfinate group is protonated while C27 is ionized was investigated. A proton was placed on the OX2 atom on the basis that the SX1–OX2 distance is longer in the ; 2DE3 crystal structure (Fig. S3A†). During the heating stage of the MD simulation, SO_2_ rotated, leading to a structure wherein the proton hydrogen bonds to the C27 sulfur (Fig. S3B†). This conformation remained throughout the simulation. When the cluster model was optimized, the proton transferred back to the sulfur, indicating that a protonated HBPS is, indeed, not a stable intermediate.

### Electrophilic aromatic substitution mechanism

**Fig. 3:**
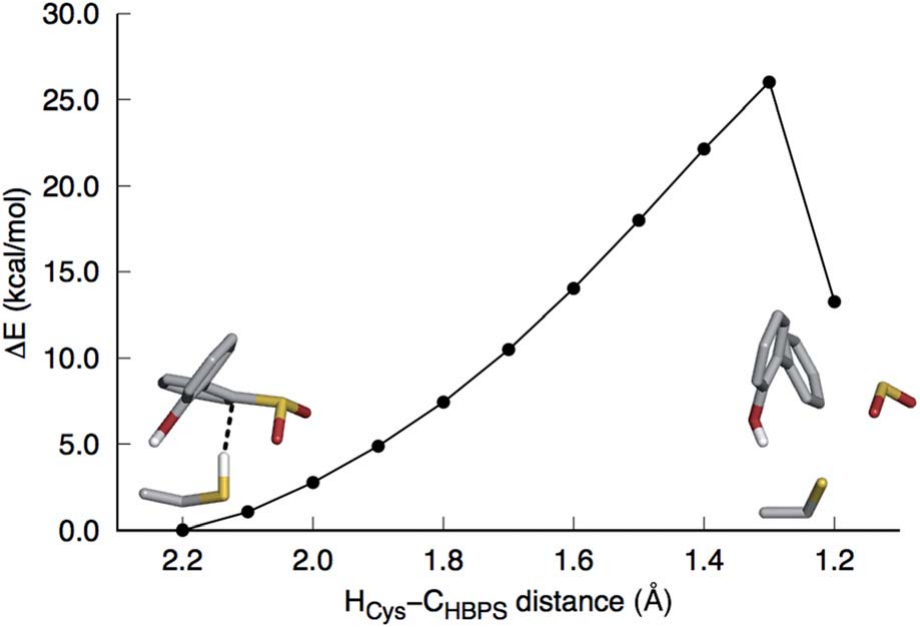
Potential energy surface scan along the H_Cys_–C_HBPS_ reaction coordinate. The model includes 2′-hydroxybiphenyl-2-sulfinate, C27, H60, R70, G73, and S25 but only the substrate and C27 are shown in stick here. The transition state is at 1.3 Å. SO_2_ is released upon formation of the C–H bond.

**Fig. 4:**
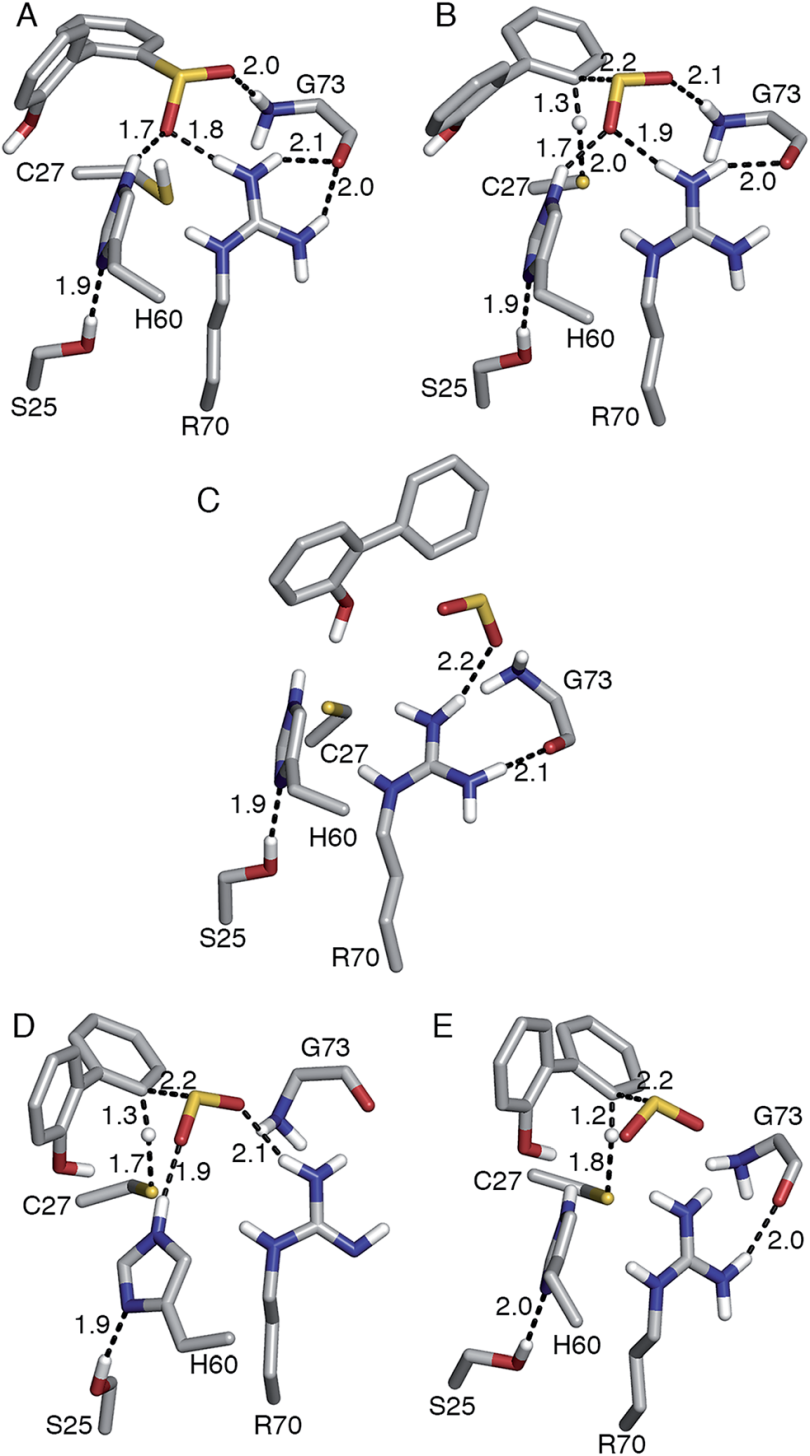
(A) Reactant, (B, D, E) transition state, and (C) product of desulfination of 2′-hydroxybiphenyl-2-sulfinate. Only polar hydrogen atoms are shown. Bond distances are in Å. Structures (A–D) were optimized at the B3LYP/6-31+G(d,p) level and structure (E) at the M06-2X/6-31+G(d,p) level. Transition state (B) and product (C) are 31.4 and 2.8 kcal mol^–1^ higher than reactant (A), respectively. Transition state (D) was modeled with R70 deprotonated at the N-η1 nitrogen.

This mechanism involves the substitution of the sulfinate group of HBPS by the C27 proton. In the MD simulation of the enzyme with neutral C27 and negatively charged HBPS, the H_Cys_–C_HBPS_ distance is predominantly around 3.1 Å (Fig. S2C†). The structure with *d*_H–C_ = 2.2 Å was chosen and scanned along the H_Cys_–C_HBPS_ reaction coordinate. The transition state is located at *d*_H–C_ = 1.3 Å with an energy of 26 kcal mol^–1^ (Fig. 3). Releasing the H_Cys_–C_HBPS_ bond constraint on both reactant and transition state and re-optimization led to a distance of 3.4 and 1.3 Å, respectively (Fig. 4A and B).

Vibrational frequency analysis confirmed the nature of the transition state and yielded a Gibbs free energy of activation (Δ*G*^‡^) of 31.4 kcal mol^–1^ (Table 1). Natural bond orbital (NBO) analysis indicates that a negative charge develops on cysteine and decreases on HBPS at the transition state (Table 2). However, intrinsic reaction coordinate calculations showed that the transition state leads directly to SO_2_ release without formation of an arenium ion (σ-complex). This desulfination mechanism follows the one-step, concerted pathway that has been recently reported for electrophilic aromatic substitution reactions such as halogenation with Cl_2_.^26^,^27^ The reaction is endothermic by 7.0 kcal mol^–1^, with a reaction free energy Δ*G*_r_ of 2.8 kcal mol^–1^. The electrophilic aromatic substitution mechanism is therefore the most feasible pathway for the desulfination of HBPS by DszB.

**Table 1:** Thermodynamic parameters (kcal mol^–1^) for desulfination of 2′-hydroxybiphenyl-2-sulfinate (HBPS) calculated at the B3LYP/6-31+G(d,p) level using different active site models of DszB

Active site model		Δ*G*^‡^	Δ*H*^‡^	–*T*Δ*S*^‡^
0	HBPS + C27 + H60 + R70 + G73 + S25	31.4	29.5	1.9
	C27S mutant	37.4	37.4	0.0
	Biphenyl-2-sulfinate substrate	30.2	27.3	2.9
	Deprotonated R70	23.1	20.9	2.2
1	HBPS + C27	31.2	30.8	0.4
2	Model 1 + H60	33.3	28.6	4.7
3	Model 1 + R70	37.8	36.1	1.7
4	Model 1 + H60 + R70	28.6	26.0	2.6
5	Model 4 + S25	29.0	25.5	3.5
6	Model 4 + G73	31.0	29.7	1.3

**Table 2:** Transition state imaginary frequencies (*ν*^‡^, cm^–1^), bond orders, and charges for Models 0–6 calculated at the B3LYP/6-31+G(d,p) level

Model	1	2	3	4	5	6	0	0^*a*^
*ν* ^‡^	966.9	865.8	827.5	657.3	662.4	574.0	553.5	697.4

**Bond order**								
S_Cys_–H_Cys_	0.53	0.52	0.47	0.43	0.44	0.42	0.42	0.47
H_Cys_–C_HBPS_	0.40	0.41	0.44	0.48	0.48	0.50	0.50	0.46

**Charge**								
HBP^*b*^	–0.50	–0.52	–0.34	–0.32	–0.32	–0.31	–0.32	–0.46
SO_2_^*c*^	–0.34	–0.34	–0.37	–0.38	–0.38	–0.38	–0.38	–0.36
Cys^*d*^	–0.37	–0.31	–0.34	–0.37	–0.37	–0.39	–0.40	–0.34
H_Cys_^*e*^	0.20	0.21	0.23	0.24	0.24	0.24	0.24	0.21
His	—	–0.04	—	–0.02	0.02	–0.02	0.02	0.01
Arg	—	—	0.82	0.84	0.85	0.85	0.86	–0.03
Ser	—	—	—	—	–0.03	—	–0.03	–0.04
Gly	—	—	—	—	—	0.01	0.02	0.00

^*a*^Deprotonated R70.

^*b*^Hydroxybiphenyl ring.

^*c*^Sulfinate substituent.

^*d*^Deprotonated cysteine.

^*e*^Cysteine proton.

### Role of active site residues

Replacing cysteine with serine in Model 0 increased Δ*G*^‡^ to 37.4 kcal mol^–1^ (Table 1, Fig. S4A†), consistent with the observed inactivity of the C27S mutant.^10^,^12^ To determine the mechanistic role of the other active site residues, Δ*G*^‡^ was calculated using different cluster models of the active site. S25 and G73 were considered for the computational model, in addition to H60 and R70. In the ; 2DE3 crystal structure, the carbonyl oxygen of G73 is hydrogen bonded to the N-η1/N-η2, while the hydroxyl hydrogen of S25 is hydrogen bonded to the N-δ nitrogen of H60 (Fig. 1A).

The activation enthalpy Δ*H*^‡^ for the different models are also compared, as the gas-phase cluster model calculations cannot provide an accurate value for the entropy contribution (–*T*Δ*S*^‡^) to the enzymatic reaction, which must account for the effects of the restriction of the reactants' motion and solvent reorganization.^28^ With only HBPS and C27 in the computational model (Model 1, Fig. S4B†), Δ*H*^‡^ is 1 kcal mol^–1^ higher than that of Model 0, but due to the more favorable entropy contribution, the Δ*G*^‡^ values are similar (Table 1). The charge distribution at the transition state is consistent with the deprotonation of cysteine and release of a neutral SO_2_ (Table 2). The addition of H60 (Model 2, Fig. S4C†) lowered Δ*H*^‡^ by 2 kcal mol^–1^ relative to Model 1 (Table 1). NBO analysis indicates that the negative charge on cysteine at the transition state decreased and was partly transferred to histidine (Table 2). However, –*T*Δ*S*^‡^ is higher, which may be attributed to rearrangement of both histidine and cysteine at the transition state in the absence of steric constraints imposed by surrounding protein residues.

On the other hand, the addition of R70 to Model 1 (Model 3) significantly increased Δ*H*^‡^ and Δ*G*^‡^ by 5 and 7 kcal mol^–1^, respectively (Table 1). The N-ε hydrogen of arginine also forms a weak hydrogen bond (2.2 Å) with the cysteine sulfur at the transition state (Fig. S4D†). There is a significant change in the charge distribution at the transition state, with the substrate aromatic ring becoming more positive as some of the charge is transferred to arginine (Table 2). With both residues in the model (Model 4, Fig. S4E†), Δ*H*^‡^ and Δ*G*^‡^ decreased by 5 and 3 kcal mol^–1^, respectively (Table 1). NBO analysis also shows that the transition state is now more product-like (*i.e.*, higher H–C bond order compared to S–H), consistent with the endothermicity of the reaction (Table 2).

The addition of S25 (Model 5, Fig. S4F†) did not reduce the barrier; however, it does appear to withdraw the negative charge from histidine at the transition state (Table 2). In contrast, the addition of G73 (Model 6) increased Δ*H*^‡^ and Δ*G*^‡^, bringing these values closer to those of Model 0 (Table 1). Glycine also forms a hydrogen bond with the sulfinate group at the transition state (Fig. S4G†). Therefore, the results suggest that among the active site residues, H60 plays the most important role in lowering the activation enthalpy to desulfination by withdrawing negative charge from C27. In contrast, the presence of R70 and G73 increased the activation enthalpy by shifting the transition state to a more product-like character.

The possibility that R70 is deprotonated was also investigated on the basis of a hypothesis that a neutral R70, together with the net negative charge at the active site due to HBPS, would elevate the p*K*_a_ of C27 (∼8), such that it would be protonated at the experimental pH (pH 8.0).^29^ The conventional wisdom is that arginine is predominantly charged in proteins, even at pH values as high as 10, because of its high intrinsic p*K*_a_ (∼12), low hydration energy, and conformational flexibility (enabling it to seek hydrogen bond acceptors).^30^ Nevertheless, there is evidence that arginine is deprotonated and acts as a general base in a few enzymes, such as inosine 5′-monophosphate dehydrogenase, pectate/pectin lyases, fumarate reductase, and l-aspartate oxidase.^31^

Aside from the proximity of positively charged residues and a hydrophobic environment, the p*K*_a_ of arginine can be perturbed by non-planarity of the guanidinium group, which would disrupt the delocalization of the positive charge over the Y-π system.^30^,^31^ In DszB, HBPS binding induces the formation of α-helices near R70 that not only limits access to hydrogen-bonding solvent, but also introduces a steric constraint that may alter the geometry of the guanidinium group (Fig. 1B). Such conditions may favor a transient deprotonated state for arginine during the catalytic reaction. Calculation of the deprotonation free energy of R70 using MD is beyond the scope of this study; however, DFT calculations were performed to determine the possible deprotonation site and effect on the reaction barrier. The cluster model used was the same one as Model 0 because the current lack of optimized force field parameters for deprotonated arginine precludes an MD simulation. The structure deprotonated at the N-η1 nitrogen was found to be more stable by 5 kcal mol^–1^ than that deprotonated at the N-ε nitrogen (Fig. S5†). The calculated Δ*H*^‡^ and Δ*G*^‡^ are 9 and 8 kcal mol^–1^ lower, respectively, while the entropy contribution is similar (Table 1). The hydrogen bond interaction of the sulfinate group with R70 and G73 are weaker at the transition state. There is an additional hydrogen bond (2.2 Å) between the hydroxyl group of HBPS and cysteine sulfur that was only previously observed when R70 was excluded from the model (*i.e.*, Models 1 and 2) (Fig. 4D). NBO analysis shows a more ‘central’ transition state, wherein S–H bond cleavage and H–C bond formation has progressed to the same extent. Compared to the model with protonated R70, the substrate aromatic ring is more negative and cysteine more positive at the transition state (Table 2). Thus, the possibility that R70 is transiently deprotonated during the reaction should be further investigated experimentally.

Finally, the role of the hydroxyl substituent of HBPS was investigated because it was observed to form a hydrogen bond with cysteine in some of the transition state models and it has been postulated to be the general base that stabilizes the transition state.^16^ However, this interaction was found to be inessential to stability, since replacing HBPS with biphenyl-2-sulfinate (BPS) in Model 0 (Fig. S4H†) actually decreased Δ*H*^‡^ and Δ*G*^‡^ slightly by 2 and 1 kcal mol^–1^, respectively (Table 1). This is consistent with the similar *k*_cat_ for the two substrates.^10^

### Solvent effects

Water molecules observed near HBPS during the MD simulation were added to Model 0 to investigate the role of water in the reaction. These correspond to WAT392 (hydrogen bonded to the sulfinate oxygen), WAT593 (hydrogen bonded to the hydroxyl oxygen), and WAT393 (hydrogen bonded to the N-η1 hydrogen of R70) in the 2DE3 crystal structure (Fig. 1A and S6†). The increase in reaction barrier for Model 7 (Table 3) indicates that WAT392 does not play a critical role in stabilizing the transition state; although, it is likely the water that hydrolyzes the released SO_2_ to bisulfite. Δ*H*^‡^ and Δ*G*^‡^ increased by as much as 6 and 3 kcal mol^–1^ for Model 9 (Table 3), respectively, possibly because the water molecules are not in an optimal orientation in the selected MD snapshot. NBO analysis also indicates that the transition state becomes more product-like with the inclusion of explicit water (Tables 3 and S1†).

**Table 3:** Imaginary frequencies (*ν*^‡^, cm^–1^), thermodynamic parameters (kcal mol^–1^), and bond orders calculated with explicit and implicit solvent at the B3LYP/6-31+G(d,p) level

Active site model		*ν* ^‡^	Δ*G*^‡^	Δ*H*^‡^	–*T*Δ*S*^‡^	Bond order	
						S–H	H–C
7	Model 0 + WAT392	351.6	33.5	33.5	0.0	0.37	0.53
8	Model 7 + WAT393	383.0	32.9	33.4	–0.5	0.38	0.53
9	Model 8 + WAT593	266.8	34.5	35.1	–0.6	0.34	0.56
	Model 0 (SMD)	712.2	33.1	31.2	1.9	0.43	0.49

A transition state closer to the gas-phase geometry and with a more moderate increase in Δ*H*^‡^ and Δ*G*^‡^ was obtained using a purely implicit solvation model (Solvation Model based on Density or SMD) (Table 3). A mixed solvation model, wherein a few water molecules are explicitly included in the model while the remainder of the solvent is treated as a continuum, was not employed in this study because of inherent pitfalls in the method, including defining the correct polarization boundary conditions between the explicit and continuum regions and evaluating the configurational entropy associated with explicit water molecules.^32^

### Choice of DFT functional

The B3LYP functional has reported limitations, including the treatment of dispersion effects.^19^ Optimization and frequency calculations for Model 0 were thus repeated using the B3LYP-D3,^33^ ω-B97XD,^34^ M06-2X,^35^ CAM-B3LYP,^36^ and MPWB1K^37^ functionals to determine which yields the most reasonable geometry and activation energy. The first three functionals include dispersion correction for a better description of hydrogen bond interactions. CAM-B3LYP was developed to correct delocalization error. MPWB1K is especially parameterized for kinetics. These functionals have been previously used to study the reactions of organosulfur compounds^38^–^40^ and proton transfer in small molecules^20^ and enzymes.^19^

M06-2X yielded the lowest Δ*G*^‡^, while MPWB1K yielded the highest (Table 4). A shorter H_Cys_–C_HBPS_ distance at the reactant state (2.6 Å) was obtained using M06-2X compared to the other functionals (3.0–3.4 Å). All functionals predict a more product-like transition state than that calculated using B3LYP. Among the dispersion-corrected functionals, only M06-2X did not yield stronger hydrogen bond interactions between the substrate and its surrounding residues at the transition state (Table 4). On the other hand, a hydrogen bond was formed between the HBPS hydroxyl group and cysteine (Fig. 4E), as in Model 0 with neutral R70. Another notable difference from B3LYP and B3LYP-D3 is the lack of interaction between cysteine and arginine, resulting in a more negative charge on cysteine and a more positive one on arginine. The negative charge on the aromatic ring is also slightly larger than that in the SO_2_ moiety (Table S2†). Overall, M06-2X is the most suitable DFT functional to model the desulfination reaction as it yields the lowest reaction barrier, a reactant structure similar to that from the MD simulation, and a transition state charge distribution consistent with the expected products.

**Table 4:** Imaginary frequencies (*ν*^‡^, cm^–1^), thermodynamic parameters (kcal mol^–1^), bond orders, and hydrogen bond distances (Å) calculated using different DFT functionals and the 6-31+G(d,p) basis set

Functional	*ν* ^‡^	Δ*G*^‡^	Δ*H*^‡^	–*T*Δ*S*^‡^	Bond order		H bond distance with SO_2_		
					S–H	H–C	His	Arg	Gly
B3LYP	553.5	31.4	29.5	1.9	0.42	0.50	2.01	1.93	2.05
B3LYP-D3	311.0	30.2	30.5	–0.3	0.36	0.55	1.96	1.87	1.99
M06-2X	429.5	28.7	27.1	1.6	0.34	0.55	2.26	1.99	2.25
ω-B97XD	244.1	33.1	31.7	1.4	0.32	0.58	1.93	1.90	1.98
CAM-B3LYP	541.1	33.1	30.5	2.6	0.40	0.50	1.98	1.94	2.01
MPWB1K	348.0	34.7	35.7	–1.0	0.35	0.54	2.04	2.37	2.09

### Kinetic parameters

Δ*G*_r_, Δ*G*^‡^, and the rate constant *k*_cat_ at the experimental optimum temperature (308.15 K (ref. 11)) were calculated using the M06-2X functional and SMD implicit solvent model (Table 5). A larger basis set, 6-311+G(3df,2p), was used for the calculation because additional polarization functions for the basis set were shown to be important in obtaining accurate geometries and energies of organosulfur compounds.^42^ However, their effect on the energies of stationary points in the DszB reaction proved to be negligible. The reaction is slightly endergonic, although it is possible that the product is more stable in an actual protein environment. The calculated *k*_cat_ is also rather low but significantly increases when a reactant structure with the H_Cys_–C_HBPS_ distance constrained to 2.2 Å (as in the MD structure) is used. The discrepancy with experimental data may be mainly attributed to the use of only one protein configuration to model the reaction and relatively small size of the cluster model. Other active site residues, such as F61, W155, and F203, which may also stabilize the transition state and product through hydrophobic interactions, were not included in the cluster model because of the significant computational cost.

**Table 5:** Gibbs free energy of reaction and activation (Δ*G*_r_ and Δ*G*^‡^, kcal mol^–1^), transmission coefficient (*κ*), and rate constant (*k*_cat_, min^–1^) for desulfination of 2′-hydroxybiphenyl-2-sulfinate by DszB calculated at the SMD/M06-2X/6-311+G(3df,2p) level

	Δ*G*_r_	Δ*G*^‡^	*κ*	*k*_cat_
Calculated^*a*^	8.6 (4.2)	30.0 (25.6)	1.4	3.0 × 10^–7^ (3.5 × 10^–4^)
Experiment				2^*b*^
				7.38^*c*^
				1.3 ± 0.07^*d*^
				1.7 ± 0.2^*e*^

^*a*^Values in parenthesis calculated using reactant with H_Cys_–C_HBPS_ distance constrained to 2.2 Å.

^*b*^ Ref. 4, pH 7.5, 303.15 K.

^*c*^ Ref. 10, pH 7.0, 301.15 K.

^*d*^ Ref. 11, pH 7.4, 308.15 K.

^*e*^ Ref. 41, pH 7.0, 303.15 K.

The calculations, nevertheless, demonstrate the feasibility of the mechanism proposed by Gray *et al.*^16^ The cluster modeling herein serves as an initial step toward generating an accurate free energy profile of the reaction by establishing the appropriate choice of reaction coordinate, protonation state of C27, and active site residues that affect the electronic structure of the transition state. This will be completed in a future work by modeling the whole enzyme using the QM/MM approach to adequately represent protein environment and employing dynamical methods (*e.g.*, umbrella sampling) to ensure that protein configurations optimum for the catalytic reaction are sampled.^43^

## Conclusions

DFT calculations therefore support an electrophilic aromatic substitution mechanism for desulfination of HBPS by DszB, reminiscent of protodesulfonation, which is a well-known reaction of arylsulfonic acids.^44^ The catalytic cysteine in DszB (C27) acts as a proton donor, unlike the case in cysteine desulfurases such as CSD, where it acts as the nucleophile.^45^ The nascent ionized C27 at the transition state is stabilized by H60, whose charge is modulated by hydrogen bond interaction with S25. The proposed electrophilic aromatic substitution mechanism is consistent with activity assays of the C27S and H60Q mutants^12^ and kinetic experiments with HBPS, BPS, and alkyl-substituted HBPS.^10^,^16^

The involvement of ionizable residues in the reaction implies that the microenvironment around the active site also plays a major role in catalytic activity.^29^ Calculations have demonstrated that the protonation state of R70 has a significant effect on the reaction barrier, which was predicted to be lower if R70 is neutral. Similarly, alteration of the local dielectric constant could explain the increased activity of the Y63F mutant (Fig. 1A).^13^ Future studies should therefore address the effect of factors including hydrogen bond interactions, proximity of charged residues, and solvent accessibility on the p*K*_a_ of the active site residues. Such comprehensive understanding of the DszB desulfination reaction is anticipated to enable prediction of mutations capable of increasing catalytic activity, thus tackling a key hurdle in the economic feasibility of industrial biodesulfurization.

## Computational method

MD simulations were performed using CHARMM version c37b1 (ref. 46) while DFT calculations were performed using Gaussian 09 Revision D.01.^47^ The crystal structure of the DszB C27S variant in complex with HBPS (PDB entry ; 2DE3) was used as the initial structure. Three systems were built to study the different reaction pathways: (1) protonated C27 and deprotonated HBPS, (2) deprotonated C27 and HBPS, and (3) deprotonated C27 and protonated HBPS. Protonation states of the other titratable residues were assigned based on p*K* values at pH 8.0 calculated using H++ and visual inspection.^48^–^51^ In particular, R70 is positively charged while H60 is protonated at the N-ε nitrogen. MD simulations were run for 2 ns using the CHARMM 36 force field for the protein^52^–^54^ and newly developed parameters for HBPS.^55^ Further details are described in ESI.†

Relaxed PES scans were performed in the gas phase at the B3LYP^56^–^58^/6-31+G(d,p) level. Initial structures were taken from the MD trajectories wherein the distance between the reacting atoms is the shortest. The cluster model includes HBPS and the C27, H60, R70, G73, and S25 side chains (truncated at Cα and saturated with hydrogen atoms) for a total of 89 atoms. Geometry optimization was performed with the Cα atom frozen to preserve the residues' positions in the protein. The reactant/product and transition state were confirmed to have zero and exactly one imaginary frequency, respectively, by vibrational frequency analysis. Intrinsic reaction coordinate calculations were also done to establish the validity of the transition state. Atom charges and Wiberg bond indices at the transition state were obtained from NBO analysis.^59^,^60^

Thermodynamic and kinetic parameters at 308.15 K were determined by single-point calculations at the M06-2X/6-311+G(3df,2p) level. Thermal corrections were obtained from vibrational analysis performed using the 6-31+G(d,p) basis set. Solvent effects were calculated using the SMD implicit solvent model.^61^ A dielectric constant of 5.6 was chosen to mimic the protein environment.^62^ The equations used to calculate the transmission coefficient and rate constant are provided as ESI.†

## Supplementary Material

Supplementary information

Supplementary information
