# Supplementary material for: Desulfination by 2′-hydroxybiphenyl-2-sulfinate desulfinase proceeds via electrophilic aromatic substitution by the cysteine-27 proton
Source: Chem Sci. 2017 May 17;8(7):5078–86. doi: 10.1039/c7sc00496f (PMC6100217; doi:10.1039/c7sc00496f)
Supplement: Supplementary file 2 [file SC-008-C7SC00496F-s002.pdf]

Electronic Supplementary Material (ESI) for Chemical Science.
This journal is © The Royal Society of Chemistry 2017

Supplementary Information

Desulfination by 2′-hydroxybiphenyl-2-sulfinate desulfinase proceeds via electrophilic aromatic substitution by the Cysteine-27 proton

Inacrist Geronimo, Shawn R. Nigam, and Christina M. Payne*

Department of Chemical and Materials Engineering, University of Kentucky, Lexington, Kentucky 40506-0046, United States

E-mail: christy.payne@uky.edu

*To whom correspondence should be addressed

1 Biodesulfurization pathway

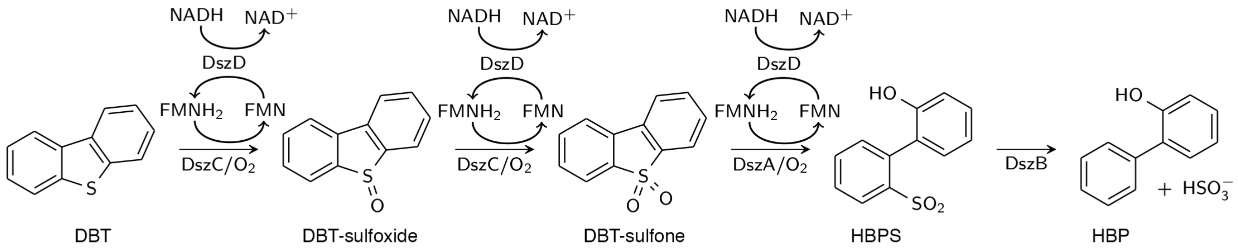

Scheme S1. Biodesulfurization pathway in *Rhodococcus erythropolis.*

Dibenzothiophene (DBT) is oxidized in two steps to DBT-sulfone by dibenzothiophene monooxygenase (DszC). One of the C–S bonds is cleaved in the third step by dibenzothiophene-5,5′-dioxide monooxygenase (DszA), yielding 2′-hydroxybiphenyl-2-sulfinate (HBPS). In the final step, HBPS is desulfinated by 2′-hydroxybiphenyl-2-sulfinate desulfinase (DszB), resulting in sulfur removal and formation of 2-hydroxybiphenyl (HBP). A fourth enzyme, the flavin reductase DszD, is required by DszA and DszC for activity.[1–3]

2 Molecular dynamics simulation

Each system was minimized for 100 steps using the steepest descent (SD) method followed by 1000 steps using the adopted basis Newton-Raphson (ABNR) method. The system was then solvated in a truncated octahedral box of TIP3P water,[4,5] with a buffer distance of 12 Å between each wall and the closest atom in each direction. Fourteen Na^+ ions (15 when both C27 and HBPS are deprotonated) were added to neutralize the system. The system size is approximately 54,000 atoms.

SD minimization of the system was performed for 1000 steps with the protein and substrate fixed. Residues within 5 Å of the substrate were released, while the substrate and remainder of the protein were restrained with a harmonic force constant of 5.0 kcal/mol/Å, and 1000 steps of SD

minimization were performed. This was followed by an additional 1000 minimization steps, with only the substrate restrained. Finally, the entire system was minimized for 1000 steps using the SD method, followed by 1000 steps using the ABNR method.

The system was gradually heated for 20 ps from 100 to 300 K in steps of 5 K every 0.5 ps and equilibrated for 100 ps. A 2-ns production simulation was performed in the NPT ensemble, using a modified Hoover thermostat[6] to maintain the temperature at 300 K and Langevin piston[7] to keep the pressure at 1 atm. Long-range electrostatics were calculated using the Particle Mesh Ewald method[8] with a 6th order b-spline, Gaussian parameter width of 0.43 Å, and mesh size of 90 x 90 x 90 Å. Van der Waals interactions were switched off between 10 and 12 Å. Bonds involving hydrogen were constrained using the SHAKE algorithm[9] and a 2-fs time step was used. The backbone atom RMSD reached a plateau at the end of the simulation, demonstrating the stability of the system (Fig. S1).

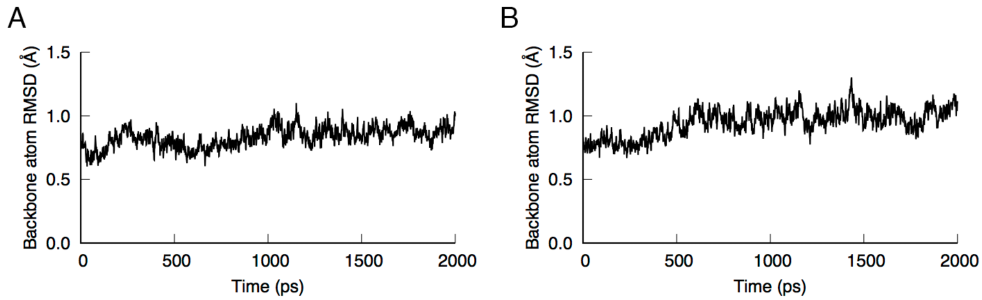

Fig S1. Backbone atom RMSD (Å) during the 2-ns production simulation of system with (A) protonated C27 and deprotonated HBPS (B) deprotonated C27 and HBPS.

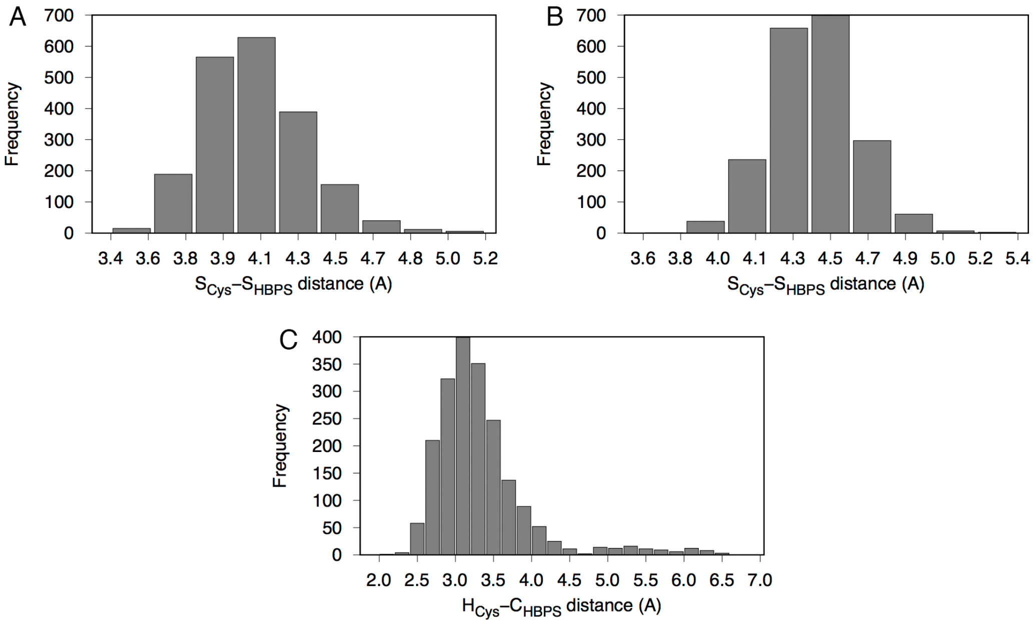

Fig S2. Histograms of (A) S_{Cys}–S_{HBPS} (protonated cysteine), (B) S_{Cys}–S_{HBPS} (deprotonated cysteine), and (C) H_{Cys}–C_{HBPS} distances calculated using 2000 snapshots from molecular dynamics simulations. The bin width is 0.2 Å.

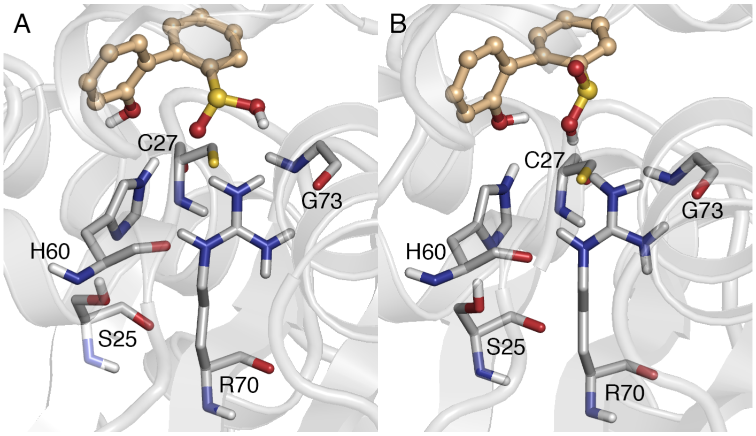

Fig S3. (A) Initial structure of DszB with bound protonated HBPS (PDB entry 2DE3[10]). The proton was placed on the oxygen with the longer S–O bond (1.50 Å). (B) MD-equilibrated structure with the proton forming a hydrogen bond with the C27 sulfur. Only polar hydrogen atoms are shown for both structures.

3 Density functional theory calculations

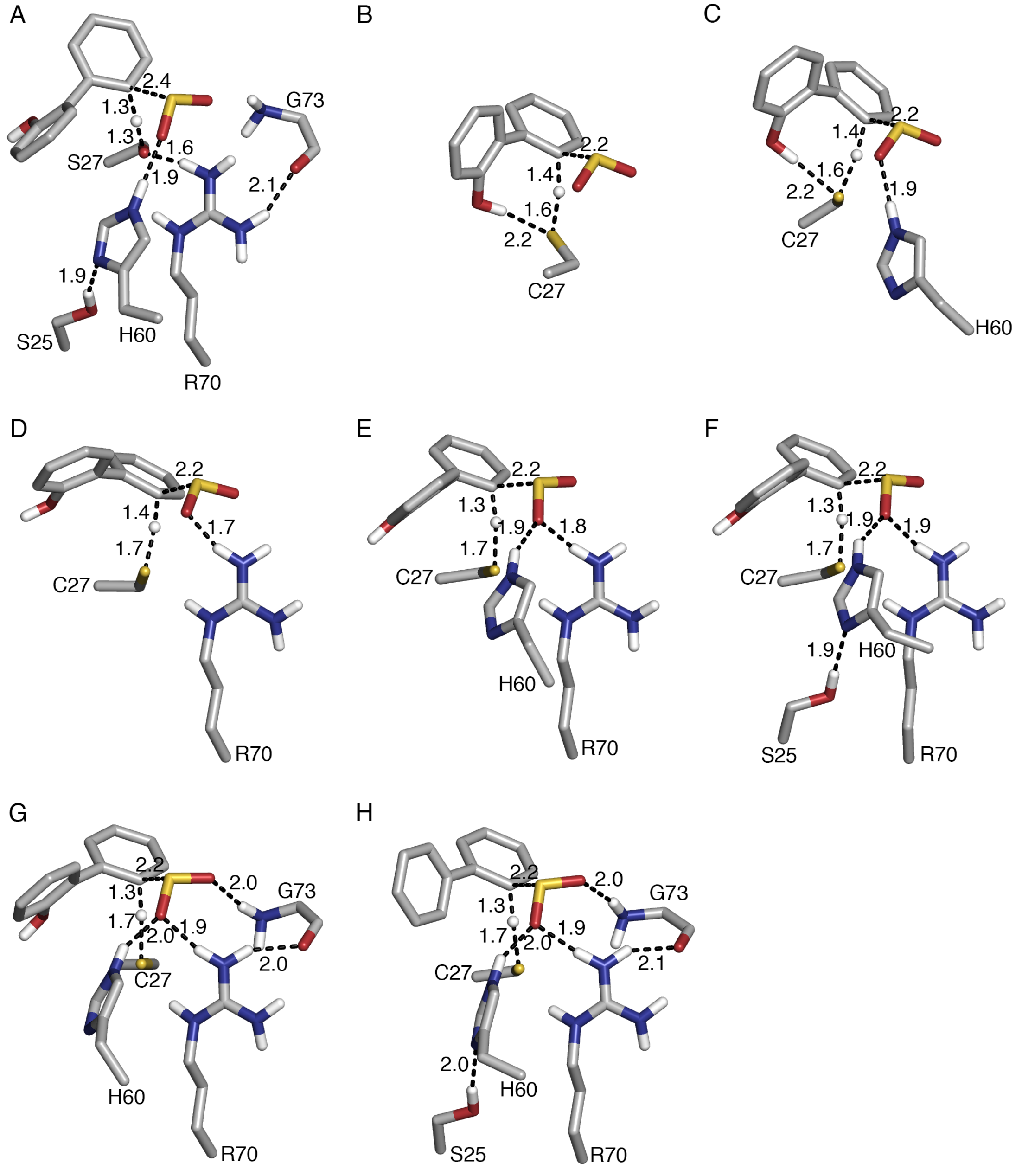

Fig S4. Transition state structures for (A) Model 0 C27S mutant, (B–G) Models 1–6, and (H) Model 0 biphenyl-2-sulfinate substrate optimized at the B3LYP/6-31+G(d,p) level. Only polar hydrogen atoms are shown. Bond distances are in Å.

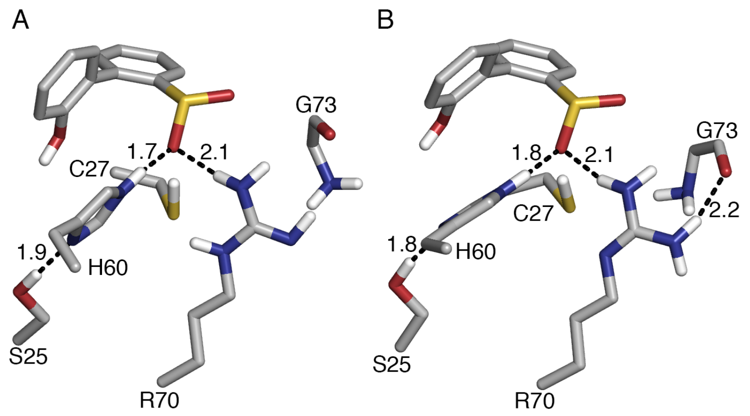

Fig S5. Reactant structure with R70 deprotonated at the (A) N-$\eta 1$ and (B) N-ε nitrogen, optimized at the B3LYP/6-31+G(d,p) level. Only polar hydrogen atoms are shown. Bond distances are in Å.

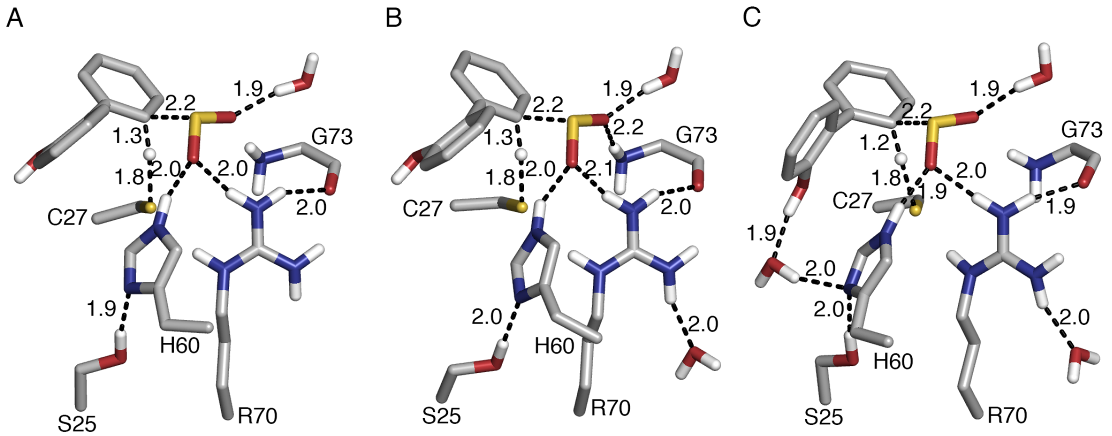

Fig S6. Transition state structures for (A–C) Models 7–9 optimized at the B3LYP/6-31+G(d,p) level. Only polar hydrogen atoms are shown. Bond distances are in Å.

Table S1. Charges at the transition state calculated with explicit and implicit solvent at the B3LYP/6-31+G(d,p) level.

Model	0	0 (SMD)	7	8	9
HBP[a]	-0.32	-0.32	-0.29	-0.30	-0.31
SO_2[b]	-0.38	-0.40	-0.40	-0.40	-0.39
Cys[c]	-0.40	-0.41	-0.40	-0.41	-0.43
H_{Cys}[d]	0.24	0.24	0.25	0.25	0.26
His	0.02	0.02	0.02	0.02	0.02
Arg	0.86	0.89	0.86	0.86	0.84
Ser	-0.03	-0.04	-0.04	-0.03	-0.03
Gly	0.02	0.03	0.01	0.02	0.02

[a] hydroxybiphenyl ring
[b] sulfinate substituent
[c] deprotonated cysteine
[d] cysteine proton

Table S2. Charges at the transition state calculated using different functionals and the 6-31+G(d,p) basis set.

Functional	B3LYP	B3LYP-D3	M06-2X	ω-B97XD	CAM-B3LYP	MPWB1K
HBP[a]	-0.32	-0.31	-0.38	-0.39	-0.38	-0.34
SO_2[b]	-0.38	-0.36	-0.31	-0.29	-0.34	-0.31
Cys[c]	-0.40	-0.43	-0.49	-0.50	-0.43	-0.49
H_{Cys}[d]	0.24	0.25	0.25	0.27	0.26	0.27
His	0.02	0.02	0.01	0.02	0.03	0.01
Arg	0.86	0.85	0.92	0.90	0.89	0.86
Ser	-0.03	-0.04	-0.01	-0.03	-0.04	-0.02
Gly	0.02	0.02	0.02	0.02	0.02	0.02

[a] hydroxybiphenyl ring
[b] sulfinate substituent
[c] deprotonated cysteine
[d] cysteine proton

4 Rate constant calculation

The rate constant was calculated using the Eyring equation[11]

$$k_{cat} = \kappa(T)\frac{k_B T}{h}\exp\left(\frac{\Delta S^{\ddagger}}{T}\right)\exp\left(-\frac{\Delta H^{\ddagger}}{RT}\right) = \kappa(T)\frac{k_B T}{h}\exp\left(-\frac{\Delta G^{\ddagger}}{RT}\right) \quad (1)$$

where R, k_B, and h are the gas, Boltzmann, and Planck's constants, respectively, $\kappa(T)$ is the transmission coefficient accounting for quantum mechanical effects, and T is the temperature. $\kappa(T)$ was calculated using the Skodje-Truhlar method[12]

$$\kappa(T) = \frac{\beta\pi/\alpha}{\sin(\beta\pi/\alpha)} - \frac{\beta}{\alpha-\beta}\exp[(\beta-\alpha)(\Delta H^{\ddagger} - \Delta H_r)] \qquad \alpha > \beta \quad (2)$$

$$\kappa(T) = \frac{\beta}{\beta-\alpha}\exp[(\beta-\alpha)(\Delta H^{\ddagger} - \Delta H_r)] - 1] \qquad \alpha < \beta \quad (3)$$

where $\alpha = 2\pi/h\nu^{\ddagger}$, $\beta = 1/k_B T$, $\nu^{\ddagger}$ is the transition state imaginary vibrational frequency, $\Delta H^{\ddagger}$ is the activation enthalpy, and ΔH_r is the reaction enthalpy (set to zero for an exothermic reaction).

References

(1) K. A. Gray, O. S. Pogrebinsky, G. T. Mrachko, L. Xi, D. J. Monticello and C. H. Squires, *Nat. Biotech.*, 1996, **14**, 1705–1709.

(2) S. A. Denome, E. S. Olson and K. D. Young, *Appl. Environ. Microbiol.*, 1993, **59**, 2837–2843.

(3) E. S. Olson, D. C. Stanley and J. R. Gallagher, *Energy Fuels*, 1993, **7**, 159–164.

(4) S. R. Durell, B. R. Brooks and A. Ben-Naim, *J. Phys. Chem.*, 1994, **98**, 2198–2202.

(5) W. L. Jorgensen, J. Chandrasekhar, J. D. Madura, R. W. Impey and M. L. Klein, *J. Chem. Phys.*, 1983, **79**, 926–935.

(6) W. G. Hoover, *Phys. Rev. A*, 1985, **31**, 1695–1697.

(7) S. E. Feller, Y. Zhang, R. W. Pastor and B. R. Brooks, *J. Chem. Phys.*, 1995, **103**, 4613–4621.

(8) U. Essmann, L. Perera, M. L. Berkowitz, T. Darden, H. Lee and L. G. Pedersen, *J. Chem. Phys.*, 1995, **103**, 8577–8593.

(9) J.-P. Ryckaert, G. Ciccotti and H. J. C. Berendsen, *J. Comput. Phys.*, 1977, **23**, 327–341.

(10) W. C. Lee, T. Ohshiro, T. Matsubara, Y. Izumi and M. Tanokura, *J. Biol. Chem.*, 2006, **281**, 32534–32539.

(11) H. Eyring, *Chem. Rev.*, 1935, **17**, 65–77.

(12) R. T. Skodje, D. G. Truhlar and B. C. Garrett, *J. Phys. Chem.*, 1981, **85**, 3019–3023.
